# Benefits and Limitations of Real‐World Patient‐Reported Toxicity Symptom Monitoring for Guidelines and Care, as Perceived by Patients, Clinicians, and Guideline Developers

**DOI:** 10.1002/cam4.70880

**Published:** 2025-04-17

**Authors:** Y. Smit, L. Verweij, A. Currie, J. J. W. M. Janssen, E. F. M. Posthuma, A. Dekker, R. P. M. G. Hermens, N. M. A. Blijlevens

**Affiliations:** ^1^ Department of Hematology Radboud University Medical Center Nijmegen the Netherlands; ^2^ Department of Internal Medicine Reinier de Graaf Hospital Delft the Netherlands; ^3^ Department of Radiation Oncology (Maastro), GROW School for Oncology, Maastricht University Medical Centre the Netherlands; ^4^ Department of IQ Healthcare Radboud University Medical Center Nijmegen the Netherlands

**Keywords:** chronic myeloid leukemia, evidence ecosystem, learning healthcare environment, patient‐reported outcome measures, real‐world evidence, toxicity monitoring

## Abstract

**Background:**

Toxicity monitoring should be modernized to include real‐world patient‐reported data. However, little is known about how stakeholders view the incorporation of real‐world patient‐reported toxicity symptoms into guidelines. This gap hinders the development of a sustained learning healthcare environment and limits the incorporation of this data into daily care.

**Methods:**

This qualitative study, reported according to COREQ, involved interviews with 29 plus 10 chronic myeloid leukemia (CML) patients and 18 CML clinicians, including eight hematologists/guideline developers. The interviews were audio‐recorded, transcribed, and independently coded in Atlas.ti. A framework, adapted from systematically sourced literature, was used for coding. Codes were assessed as either beneficial or limiting. An expert panel of all CML guideline developers completed and prioritized the identified knowledge gaps through a RAND‐modified Delphi procedure.

**Results:**

Thirty‐one benefits and limitations of systematically monitoring patient‐reported toxicity symptoms in the real world were identified. Compared to an existing framework, novel benefits centered around the use of aggregated data: Participants viewed real‐world patient‐reported toxicity symptoms as a way to systematically include patients' toxicity symptoms in the guidelines; personalize guideline advice; and fill knowledge gaps. The expert panel agreed on 14 knowledge gaps in chronic myeloid leukemia care that could be addressed through such data. Novel limitations focused on the suitability, acceptance, and applicability of toxicity symptom monitoring in routine clinical practice. Participants felt that this monitoring does not establish a causal link between medication and symptoms, and it has no added value over open conversation.

**Conclusions:**

The benefits and limitations of adopting patient‐reported real‐world toxicity symptom monitoring need to be leveraged and addressed to ensure maximum value and uptake. Guideline developers viewed aggregated data as beneficial. The identified knowledge gaps provide concrete points of action for CML guideline development.

## 1 Introduction

The routine monitoring of patient‐reported symptoms, with feedback to healthcare professionals, has improved both quality of life and survival in solid oncology patients [1, 2, 3, 4]. This evidence firmly establishes patient‐reported outcome measures: Monitoring across the care continuum is now recommended by ESMO to detect and manage treatment toxicities early, as well as relapses, and tailor care [5]. In hematooncology, however, evidence for symptom monitoring is still limited [6]. Nevertheless, there is a need to modernize toxicity assessment by incorporating patient‐reported outcomes into real‐world care in this field as well [7, 8].

In addition to improving the quality of care, patient‐reported toxicity symptom assessment can be systematically aggregated and evaluated as a quality metric [5]; contribute to the scientific debate; and inform the development of evidence‐based guidelines. At present, hematooncology guidelines rely almost exclusively on clinical trials. However, these trials rarely incorporate patient‐reported outcomes [7] and their selective inclusion criteria limit real‐world applicability.

Patient‐reported outcomes are particularly relevant in chronic conditions like chronic myeloid leukemia (CML), where most patients require lifelong tyrosine‐kinase inhibitors (TKIs). TKI use is associated with substantial low‐grade toxicity [9], which is often underestimated by clinicians [10]. Approximately 30% of patients require a TKI switch due to intolerance [11]. If CML guidelines focus too heavily on survival and disease control, they risk losing relevance. For most patients, disease control is adequate, making toxicity management the primary concern.

So, why are CML patient‐reported toxicity symptoms not collected at an aggregated level and used to guide CML care. As a first step, CML guideline developers need to assess whether monitoring CML patient‐reported toxicity symptoms is useful for the evolving CML guideline, and if so, in what way?

At the same time, the question remains: How should real‐world TKI‐related toxicity symptoms in CML patients be measured? Patient‐reported symptoms do not necessarily imply that the TKI used caused the symptoms (toxicity), much like symptoms or adverse events reported by professionals do not imply causality. The establishment of a patient‐reported Common Terminology Criteria for Adverse Events (PRO‐CTCAE) by the National Cancer Institute shows that adverse events and tolerability reported by patients are now considered valuable sources of information on treatment toxicity [12].

In CML, we know that patients report worse symptoms such as depression, dyspnea, fatigue, pain, and composite symptom‐burden scores (e.g., nausea, diarrhea, itching, skin changes, and swelling of arms or legs) compared to controls or the general population [13, 14]. Since the CML itself is well controlled in the vast majority of patients, the most plausible explanation at the group level is the TKI treatment. However, no patient‐reported instrument currently has sufficient content validity to measure TKI‐related toxicity symptoms [15].

The development of a new instrument needs to consider content validity, feedback on results, and a sustainable workflow [16]. However, these factors alone do not guarantee successful uptake [4]. The views of intended users can create barriers and facilitators for uptake, and these need to be addressed from the outset. While extensive knowledge is available on the perspectives of end‐users of patient‐reported outcomes in general, there is little focus on toxicity symptom reporting by patients in particular [17, 18, 19, 20, 21, 22, 23, 24, 25, 26, 27, 28, 29, 30, 31, 32, 33, 34, 35, 36, 37, 38, 39, 40, 41]. Perspectives from the field of hematooncology are limited, do not focus on toxicity symptom reporting, and reveal specific limitations [23, 42, 43, 44, 45].

To prepare care and guideline development for real‐world toxicity symptom monitoring, we sought the views of patients, clinicians, and clinician‐guideline developers. What do they see as the benefits and limitations of real‐world toxicity symptom monitoring, and how do they view the use of its aggregated data in individual care and guideline development? To pave the way for the actual use of such data in CML guideline development, we also identified knowledge gaps in CML care that could be addressed through systematic patient‐reported toxicity symptom monitoring.

## 2 Material and Methods

### 2.1 Design and Setting

This qualitative study interviewed CML patients and professionals, reported in accordance with the COREQ checklist [46] (Table S1 in the Data S1), and included a Delphi procedure. We chose a qualitative design to capture the full spectrum of views on the uptake of real‐world TKI‐related toxicity symptom monitoring and to address knowledge gaps. Ethical approval was waived by the institutional Medical Ethical Committee, as the study was not subject to the Medical Research Involving Human Subjects Act.

The qualitative interviews were conducted as part of the development and validation phases of an instrument designed to capture real‐world patient‐reported TKI‐related toxicity symptoms, hereafter referred to as the test instrument, described in Table S2 in the Data S1. A graphical overview of patients' scores was included as part of the instrument.

Dutch CML patients are treated in eight academic hospitals and 68 general hospitals [47]. In order to give them the tools to manage and monitor their own CML, interpret BCR::ABL1 results, and act on them, the nationwide digital care platform CMyLife was established. CMyLife includes a patient portal (www.cmycml.nl) and a guideline application [48, 49]. Currently, patients on the platform have access to their own electronic personal health environment, which bridges individual hospitals' electronic patient files to the nationwide CMyLife guideline application. The test instrument is intended to function within the CMyLife digital care platform.

**FIGURE 1:**
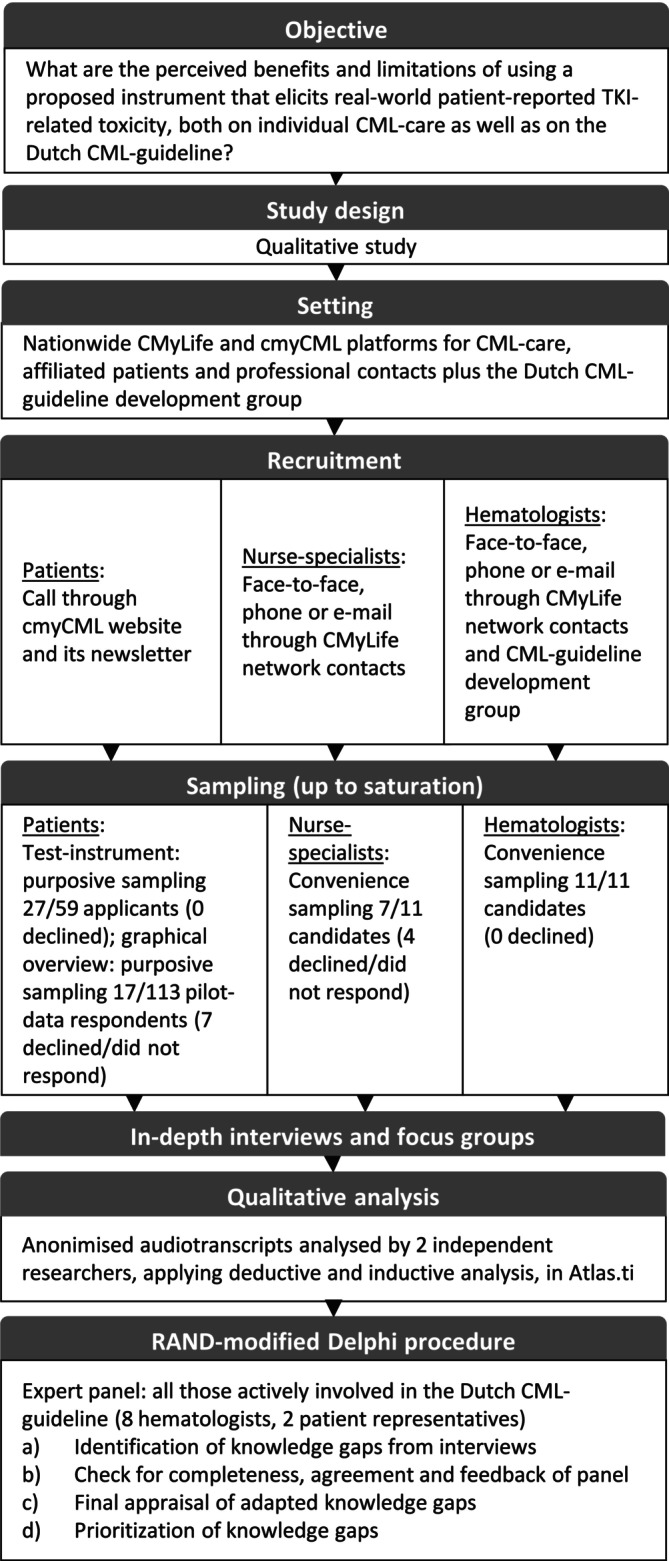
Flowchart of the study.

The Delphi procedure was conducted within the national HOVON (Dutch–Belgian Cooperative Trial Group for Hematology‐Oncology) CML‐MPN working group. This group is the guideline development group for the Dutch CML guideline [50]. Figure 1 summarizes the study flow.

### 2.2 Interviews

#### 2.2.1 Recruitment

Recruitment and sampling took place during two phases: (1) the qualitative development and validation phases; and (2) the subsequent quantitative validation phase of the test instrument.

In the first phase, 27 out of 59 patients who responded to a call on the CMyLife platform were purposefully sampled (up to data saturation) to reflect differences in sex, age, time since diagnosis, education, and hospital type. Hematology nurse‐specialists (in training) were recruited through our network via convenience sampling, and additional hematologists were recruited until saturation was reached.

In the second phase, a further 10 patients were recruited for interviews based on the graphical overview constructed from their own data. These 10 patients were sampled from a group of 113 patients who responded to another invitational call on the CMyLife platform and participated in pilot data collection with the test instrument for 6 months (quantitative validation phase). The sampling was purposefully done to reflect differences in sex, age, time since diagnosis, current treatment, and completion of pilot data.

Lastly, all Dutch hematologists actively involved in CML guideline development within the national HOVON CML‐MPN working group were recruited for the Delphi procedure.

#### 2.2.2 Sessions

Two researchers (Y.S. and A.C.) conducted focus groups and individual interviews, hereafter referred to as sessions, using semi‐structured interview guides centered on perceived (dis)utility (Data S1, 7). Two to 5 days prior to the sessions, the test instrument and/or graphical overview were sent to participants, asking them to either fill it in (patients) or read it through (professionals and patients for their own graphical overview). Informed consent was obtained before the sessions began, and all sessions were audio recorded, transcribed, and anonymized.

### 2.3 Analysis

We systematically reviewed the literature and extracted (sub)themes from a recent comprehensive systematic review [17] completing these themes with codes from other systematic reviews plus more recent primary studies [18, 19, 20, 21, 22, 23, 24, 25, 26, 27, 28, 29, 30, 31, 32, 33, 34, 35, 36, 37, 38, 39, 40, 41]. We used this framework as a code tree for deductive coding (Table S3 in the Data S1). When no suitable codes were available, we applied new codes through inductive coding. We then categorized codes according to preexisting (sub)themes or placed them in new categories when existing themes did not apply. If necessary, themes were rearranged to better reflect our data. We labeled subthemes as a benefit, limitation, or mixed. Codes, (the arrangement of) themes, and benefit/limitation labeling were discussed between two researchers, who initially coded independently until consensus was reached.

### 2.4 RAND‐Modified Delphi Procedure

The expert panel consisted of 10 members: eight hematologists and two patient representatives from the patient organization Hematon. This group included all hematologists actively involved in the Dutch CML guideline as part of the HOVON CML‐MPN working group. The procedure consisted of four rounds (described in detail in the Data S1, 10): (1) identification of knowledge gaps from the interviews; (2) verification of completeness of, and agreement with, the identified knowledge gaps, followed by adjustments if needed; (3) appraisal of the set; and (4) rating of priorities.

## 3 Results

### 3.1 Interviews

We interviewed 27 patients, plus 10 additional patients, seven nurse‐specialists (one in training), and 11 hematologists, eight of whom were involved in CML guideline development. Participants' characteristics are described in Table 1. Compared to the existing evidence framework [17, 18, 19, 20, 21, 22, 23, 24, 25, 26, 27, 28, 29, 30, 31, 32, 33, 34, 35, 36, 37, 38, 39, 40, 41], four new benefits, two new limitations, and three new mixed benefit/limitation themes emerged (Table 2). An overview of these new subthemes with illustrative quotations is provided in Table 3 (other subthemes can be found in Table S4 in the Data S1). Here, we describe the new subthemes, as well as those that emerged with an additional focus compared to the existing evidence. Table S3 in the Data S1 provides an overview of the differences between the existing evidence and this study.

1. Active Patient Involvement and Partnership

**TABLE 1:** Characteristics of participants in sessions.

**Patients (*n* = 27)** ^a^ **Test instrument, including prototype graphical overview**	
Female (%)	16 (59%)
Mean age (range)	59 years (33–77 years)
Mean time since diagnosis (range)	6 years (0–16 years)
Treatment hospital	University medical center: 9 (30%) General hospital: 17 (70%)
Education level	Low educated: 1 (4%)
	Middle educated: 3 (11%)
	High educated: 19 (70%)
	Unknown: 4 (15%)
Current treatment	Not available
**Patients (*n* = 10)** ^a^ **Graphical overview own pilot data**	
Female (%)	6 (60%)
Mean age (range)	60 years (47–84 years)
Mean time since diagnosis (range)	7 years (3–10 years)
Treatment hospital	Not available
Education level	Not available
Current treatment	Asciminib (1), bosutinib (1), dasatinib (3), imatinib (3), nilotinib (1), and ponatinib (1)
**Nurse‐specialists (in training) (*n* = 7) Test instrument, including prototype graphical overview**	
Female (%)	7 (100%)
Hospital	University medical center: 3 (43%)
	General hospital: 4 (57%)
**Hematologists (*n* = 11) (8 of whom guideline developers) Test instrument, including prototype graphical overview**	
Female (%)	6 (55%)
Hospital	University medical center: 6 (54.5%)
	General hospital: 5 (45.5%)
Involved in Dutch CML guideline development	8 (72%)

^a^ These groups may have overlapped to some extent.

**TABLE 2:** Framework of perceived benefits and limitations of real‐world patient‐reported TKI‐related toxicity symptom monitoring, arranged according to five themes that emerged after qualitative analysis of sessions with CML patients, nurse‐specialists and hematologists/guideline developers^a^.

Active patient involvement and partnership
Enables greater awareness and reflection (Benefit) *Objectifies subjective experience (Benefit)*
Encourages patient involvement (Benefit)
Facilitates goal setting and shared decision making (Benefit)
Influences honesty (Limitation)
Permits discussion of sensitive topics (Benefit)
Enables *self‐prevention* and self‐care (Benefit)
Leads to worse symptom experience (Limitation) Fulfills desire to help others (Benefit)
2 Focus of consultation
Helpful as a screening tool (Benefit)
Prioritizes patients' needs (Benefit)
Provides reassurance that clinicians care (Benefit)
Provides one piece of the picture (Limitation)
Structures consultations and improves efficiency (Benefit)
Provides redundant information (Limitation) *Suits paramedical consultation (Mixed)*
3 Quality of *individual patient* care
Helps determine *and monitor* side effects of treatment (Benefit)
Prompts appropriate, standardized action *and acceptance* (Benefit)
Assists in learning (Benefit)
Can inaccurately estimate the problem/not specific enough to be clinically meaningful (Limitation)
*Does not establish a causal link (Limitation)*
5 Suitability and acceptability
Suitability for all patients, *clinicians, and workflows (Limitation)*
Confronts too much with disease (Limitation)
*Usefulness depends on (change in) symptom severity (Mixed)*
*Has no added value over open questions or conversation (Limitation)*
6 Improving care *and clinical guidelines* with aggregated data
*Real‐world data has pros and cons (Mixed)*
Benchmarks clinical trials and medication costs (Benefit)
Puts individual experience in context (Benefit)
*Systematically includes patients' experiences in guidelines (Benefit)*
*Personalizes guideline advise (Benefit)*
*Fills in knowledge gaps (Benefit)*

^a^ Newly identified themes, subthemes, and foci compared to previous studies [17, 18, 19, 20, 21, 22, 23, 24, 25, 26, 27, 28, 29, 30, 31, 32, 33, 34, 35, 36, 37, 38, 39, 40, 41] *in italics*.

**TABLE 3:** Novel themes and subthemes^a^ with an assessment of benefit or limitation, with illustrative quotes.

Themes and subthemes	Benefit or limitation	Illustrative quotes
**Active patient involvement and partnership**		
*Objectifies subjective experience*	Benefit	It [graphical overview] does somewhat reflect how you feel, how you experience the side effects. Because you can explain it, but then he can also see it in the charts how things are (P) You can see it. It clearly says there are symptoms. In any case, it's not a figment of the imagination. I find that difficult sometimes (P)
Enables *self‐prevention* and (self‐)care	Benefit	And so, I also know that with this medicine, at least for me actually, I might need to increase my fluid intake a lot compared to before 2004, so I have fewer muscle cramps when exercising (P) But I just think, I keep finding it interesting for myself that I can keep control where I can, also uhm the prevention of side effects (P)
**Focus of consultation**		
*Suits paramedical consultation*	Mixed	Yes, the doctor is indeed mainly concerned with, uhm, is the disease well suppressed, uhm, and a little bit about quality of life, but yes, not very extensively, because that is not the core business [P] So that is the nurse specialist, and uh who asks the questions, looks at where the scores are high and sees whether she can come up with something herself to alleviate that symptoms or uh or consult with the pharmacy or consult with us, what can we do about that (H/GD) But they [toxicity symptoms] are sometimes not important enough to discuss, for various reasons. Because you don't need it at the moment, or because you as a doctor can't do anything about it, and because as a doctor you are trained to screen for side effects that require action
**Quality of *individual patient* care**		
Helps determine *and monitor* side effects of treatment	Benefit	And uhm yes, it also seems pretty important to me to keep track of, and especially for new patients, so they know that, yes, gosh, in the beginning it can be very intense, it can become less or there can be symptoms that only occur after a few months (P) So, I can imagine that it's nice to see this [test] instrument every three, four months of that patient. Then I can compare them and then I think, well, this has remained stable with this patient. But if he suddenly says he cannot walk three meters anymore, then I have a reason to pick that out, you know (NS) Because you have patients that couldn't take anything, and then I put them on this new medication, you know, an unregistered medication, asciminib for example, and then I want to know how that works and what the impact is (H/GD) For example, there is a side effect and that may be a reason to switch treatment, and then you can see, look, just like that in a score, look, indeed, that switch did work or not (H/GD)
Prompts appropriate, standardized action *and acceptance*	Benefit	Not all problems can be resolved of course, but if you know where things come from, then you can interpret, uhm, more easily, uhm, for yourself, I mean, oh, okay, you know, I don't have to worry that I feel this, I don't have to worry that I have that (P) With a lot of side effects is not about the doctor's action at all, but to be heard, to get advice on how to deal with them in life, to have, uhm, named them, to get confirmation that they are non‐lethal (H/GD) If the patient thinks: “Okay, it's not from the disease, okay, I don't have to do anything about it, well, then I'll see how I deal with it” (H/GD)
*Does not establish a causal link*	Limitation	Like, yeah, where does it come from. And that is very difficult. Yes. And then again, sometimes I think, eh, I have a lot of night sweats, and then you think: is it the menopause or not. But me, yes, that has never bothered me that much, but yes, I am just soaking wet at night [P] The problem I have is that sometimes I no longer know where the complaints come from. Is it a side effect of the drug, is it from the CML? I also have a hernia … [P] Complaints or side effects also occur with other diseases, I did not really know what to complete […] I try to distinguish what is due to CML and what is due to another disease [P]
**Suitability and acceptability**		
Suitability for all patients, *clinicians, and workflows*	Limitation	[…] because not everybody has a computer, so yes, then you'll need old fashioned paper, or an app (NS) We have also started with stand‐alone applications, uhm, in which you, uhm, set out these kinds of questionnaires outside your own electronic health record environment and visualize them. This has enormous disadvantages, because people must download separate apps for this, we often cannot see it, it is not part of your workflow if you have your electronic patient file open in your outpatient clinic (H/GD) If you want to implement it nationally with four different electronic health record systems and 80 different ways of implementation, I'm curious if that will work (H/GD)
*Usefulness depends on (change in) symptom severity*	Mixed	Well, I don't have any side effects, so yes, the side effects I have then I think: that has to do with my age, I'm getting older. Uh, I don't know what to keep track of either […] no, [I] do not [want to] keep track (P) I feel that this questionnaire does not apply to me now. It did how it was, but not how it is now (P) Yes and well, that's my personal opinion, that it especially helps those people with a lot of complaints (H/GD) Well, especially when starting with those pills. So, the first three months I would be more focussed on that list (H/GD)
*Has no added value over open questions or conversation*	Limitation	An open question, that that would work better (P) Well, why should a patient need a lead to go to a doctor with his complaints? Because I think, if you have a complaint or a symptom or a side effect of a medication or whatever […]. You also always have your outpatient visits or contact with your doctors […] and then you can just discuss that. I don't really need a proof for that, let's put it that way (P) So, I find that difficult. What does such a questionnaire do, which you cannot simply pick up in a conversation? (H/GD)
**Improving care *and clinical guidelines* with aggregated data**		
*Real‐world data has pros and cons*	Mixed	Uhm, that's the Achilles' heel of real‐world research, right that, uhm, that data are collected in a less structured manner. That makes it really very difficult to draw reliable conclusions, other that general patterns (H/GD) […] and there are so many snapshots that due to the large number of measurements you make, overall common denominators can still be distilled (H/GD) […] gives information in patients with an enormous diversity of variables that have not become visible in normal guideline development … uhm … gives information about multi‐drug use, because it is not only the TKI, but it is also the combination with treatment that can give or reduce or worsen symptoms, so that interaction (H/GD) So that means that uhm the standard guideline contains data of short duration, observations from studies mainly, occasionally from observational studies. Collecting patient‐reported outcomes for guideline use provides information about real‐world (H/GD)
*Systematically includes patients' experiences*	Benefit	It is just the value of experience […] the fact that we uhm familiarize uhm ourselves with what patients experience […] I call it patients' knowledge that needs to be brought in to the uhm, public domain (H/GD)
*Personalizes guideline advise*	Benefit	[…] and that is personalizing, right? Of treatment choices based on the guideline. So that personalization, uhm, that's often based on side effects or risk profiles, but here we are talking side effects. So, if we know better what bothers patients, yes, that, that would, well, yes, you could make different therapy choices so to speak (H/GD) And for guidelines, look, whatever we're developing now is decision aids. So, if you choose a certain strategy treatment or something, when, yes, what trade‐offs do you make? Yes, it's important for that too. […] Whether this medicine affects sex quality or temperature sense, just to mention 2 categories. […] The guideline does not go that deep. The guideline is still very much focused on effectiveness. […] That requires a considerable cultural change (H)
*Fills in knowledge gaps*	Benefit	[…] the solutions for those side effects. Because that is another thing, […] because now it is more about signalling, recognizing complaints, actually. One [care professional] says: go to a physiotherapist, the other gives you a pot of magnesium, a jar of calcium, well, what can be done about it? And what options are there? (P) Well, I think that there are unanswered questions. Things were we, that we don't know well yet. […] for example, I don't know, say, I have a patient on imatinib, and then I switch to uhm a second generation TKI […], what are the chances that the symptoms disappear? (H/GD) Can you expect something when you lower the dosage? (H/GD) I can hardly imagine that this would really have an impact on the clinical guideline. […] there is also quite a bit of literature about side effects in general, in real‐world registries […] the patterns are clear to us, but the extent is really very difficult to evaluate reliably [enough to decide] that I have to take that or that medicine (H/GD)

^a^ Novel findings compared to previous studies [17, 18, 19, 20, 21, 22, 23, 24, 25, 26, 27, 28, 29, 30, 31, 32, 33, 34, 35, 36, 37, 38, 39, 40, 41] *in italics*. Identifiers in brackets after quotes: H/GD: hematologist/guideline developer; NS: nurse‐specialist; P: patient.

Within this theme, the new subtheme, “Objectifies subjective experience,” emerged from patients who expressed that the graphical overview can clarify and strengthen their perspective, enhancing their confidence in discussing their symptoms with professionals and peers. Additionally, an further focus emerged: patients mentioned the use of self‐prevention techniques to prevent symptoms, such as increasing fluid intake before exercise. Based on this, we renamed the subtheme to: “Enables self‐prevention and self‐care.”

2 Focus of Consultation

Within this theme, an additional focus, “Suits paramedical consultation,” emerged, which combined two previous subthemes (“Shifts away from the main medical problem” and “Raises unrealistic expectations for care”) were merged. Patients acknowledged that quality of life issues are not typically the core responsibility of medical doctors, and hematologists admitted that they do not always discuss toxicity symptoms with patients, especially if no medical action is required. Hematologists viewed toxicity symptoms as more within the realm of nurse‐specialists, and patients shared positive experiences with nurse‐specialist consultations on toxicity symptoms. When a nurse‐specialist is available, shifting responsibilities can benefit the focus of consultation of hematologists. However, if no nurse‐specialist is available, this shift in focus may be a limitation for hematologists' consultations, as it diverts attention from the main medical issue.

3 Quality of Individual Patient Care

Within this theme, an additional focus emerged. Both patients and professionals found that the test instrument facilitated the acceptance of symptoms that cannot be resolved or are unlikely to improve. Understanding that certain symptoms were related to TKI toxicity rather than the CML itself helped patients move forward. As a result, we renamed the subtheme “Prompts appropriate, standardized action *and acceptance*” to better reflect the role of acceptance in this process.

Additionally, the subtheme “Does not establish a causal link” emerged. Patients expressed uncertainty about whether their symptoms were caused by CML medication or other factors, such as the CML itself, aging, or other diseases or medications. Some patients self‐censored symptoms they attributed to other causes.

4 Suitability and Acceptance

Patients mentioned that not all clinicians were digitally skilled enough to work with the test instrument. Professionals mentioned that stand‐alone applications have disadvantages, as they require separate installations and do not integrate with the hospital workflow. We therefore renamed the subtheme “Suitability for all patients” to include clinicians and workflow considerations.

Moreover, two new subthemes were identified, First, some patients felt the test instrument did not apply to them because they either did not experience symptoms or attributed their symptoms to other causes, such as aging. The instrument and graphical overview were considered most useful for severe symptoms or changes in symptoms, for example, at the start of a (new) therapy. This led to the subtheme “Usefulness depends on (change in) symptom severity” (mixed benefit and limitations).

Additionally, some patients and professionals felt that open‐ended questions or conversation were more effective and that the test instrument had no added value. This resulted in a new subtheme: “Has no added value over open questions or conversation” (limitation).

5 Improving Care and Clinical Guidelines With Aggregated Data

Within this theme, an additional focus and three new subthemes emerged. As an additional focus, hematologists/guideline developers stated that real‐world patient‐reported data could provide diverse and long‐term insights, including information on drug–drug interactions, data that can only be gathered from real‐world settings. However, they also noted that real‐world data are collected in a less structured manner compared to clinical trials, making it more difficult to draw reliable conclusions. Despite this limitation, they acknowledged that large datasets could still reveal meaningful patterns, leading to the subtheme “Real‐world data has pros and cons” (mixed benefit/limitation).

Another subtheme emerged from the recognition that patient‐reported data systematically provide insight into patients' experiences, which was considered valuable in itself. This led to the new benefit: “Systematically includes patients” experiences in guidelines'. Building on this, hematologists/guideline developers suggested that the test instrument has the potential to “Personalizes guideline advise” and “Fills in knowledge gaps” (new benefits). Identified knowledge gaps with the potential to be addressed by the test instrument were incorporated into the Delphi procedure.

Though patients were not specifically interviewed about the use of aggregated data, they expressed a strong interest in learning from it. They wanted to know how to prevent and/or mitigate symptoms—an important perspective which was not addressed by professionals. As a result, this knowledge gap was also included in the Delphi procedure.

### 3.2 RAND‐Modified Delphi Procedure

In the first round, seven out of 10 expert panel members responded. They gave feedback on the completeness of nine knowledge gaps identified from the interviews. Their input led to the addition of five new knowledge gaps.

In the second round, seven respondents approved of the revised set. In the third round, the 14 knowledge gaps were prioritized based on their relevance to the Dutch CML guideline (Table 4). The knowledge gap “What is the burden of single toxicity symptoms, and over what time period do toxicity symptoms appear and/or disappear?” was ranked as the highest priority by five out of 10 expert panel members involved in the prioritization process.

**TABLE 4:** Knowledge gaps in CML care that can be solved (in part) by real‐world patient‐reported toxicity symptom monitoring, as identified and prioritized by the Dutch CML guideline working group and patient representatives.

Order of priority	Knowledge gap
1	Per TKI: what is the burden of single toxicity symptoms, and over what time period do toxicity symptoms appear and/or disappear?
2^a^	How many patients switch from which TKI, due to intolerability, and for what symptoms?
2^a^	To what extent do patients and clinicians recognize TKI‐related toxicity symptoms, and can standardized instruments help recognize TKI‐related toxicity?
4	Which toxicity symptoms change in severity after a change in TKI dosage, or remain equal or newly appear, and by how much?
5	Which toxicity symptoms change in severity after switching from another TKI, or remain equal or newly appear, and by how much?
6	How can toxicity symptoms be prevented and/or alleviated?
7	Which toxicity symptoms change in severity after stopping TKI treatment, or remain equal or newly appear, by how much, and over what time period?
8^a^	To what extent does TKI‐related toxicity influence medication adherence by CML patients?
8^a^	To what extent is the burden of toxicity symptoms affected by patient‐ and (co)medication factors?
10^a^	Which toxicity symptoms change after restarting TKI therapy, or newly appear, and by how much?
10^a^	Per TKI: how does disease response (BCR::ABL1 control) weigh against tolerability?
12	How much does food in combination with TKI intake influence toxicity symptoms, as experienced by patients?
13	What is the relationship between TKI plasma concentration and experienced toxicity symptoms? How useful are TKI plasma concentration measurements in guiding TKI dose reduction?
14	What is the effect of growth factors because of hematological toxicity on toxicity symptoms?

^a^ Ex aequo.

## 4 Discussion

This study explored stakeholders' perspectives on capturing real‐world patient‐reported TKI‐related toxicity symptoms using a pilot instrument. From these insights, we developed a comprehensive framework outlining the perceived benefits and limitations for both care and guideline development. While many viewpoints aligned with existing literature on patient‐reported outcomes in general, we identified nine new benefits and/or limitations, along with four extensions to previously recognized ones. Here, we discuss the implications for clinical care and guideline improvement in CML and beyond.

Three newly identified limitations related to “Suitability and acceptability.” Future efforts should address these limitations, for example, by incorporating a single overarching question to filter out patients who do not experience symptoms or prefer not to participate. While such approaches require validation, they could help prevent overestimation at the group level. Notably, concerns were raised about the digital skills of both patients and professionals, as our approach relies on a digital care platform. This platform offers the advantage of directly involving patients while bypassing siloed and unstandardized provider data. However, its limitation lies in its purely digital workflow, which operates outside the hospital's usual digital environment. To avoid inclusion bias, it is essential to support less digitally skilled patients in participating, possibly through non‐digital alternatives. Additionally, ensuring the ongoing engagement of professionals requires integrating the platform within their hospital's digital workflow.

To maximize the value of TKI‐related toxicity symptom monitoring, collaboration with both patients and professionals during instrument development is essential. Identified benefits should be leveraged through strategies that inform, motivate, and support users. These efforts can also help persuade nonusers of the benefits they may not yet recognize.

Two extensions to the framework of existing studies [17, 18, 19, 20, 21, 22, 23, 24, 25, 26, 27, 28, 29, 30, 31, 32, 33, 34, 35, 36, 37, 38, 39, 40, 41] centered on further patient empowerment. First, patients indicated that discussing toxicity symptoms helped them accept symptoms that could not be alleviated. While professionals expressed frustration over their inability to provide a solution, patients reported that simply understanding their symptoms helps them move forward.

Second, patients wanted to identify symptom patterns to better prevent or manage their symptoms. Some were already tracking their symptoms, reflecting characteristics of so‐called “e‐patients” (equipped, enabled, empowered, and engaged patients) or even “expert‐patients” (those seeking deeper understanding and participation in science). This highlights a desire for self‐management that goes beyond what current healthcare practice typically offers [51, 52].

It is important to note that, at the individual level, reported symptoms do not imply causality—a limitation acknowledged by participants. However, consistently collected and standardized real‐world patient‐reported toxicity data are considered unique and valuable for toxicity characterization in hematooncology [7].

Most importantly, we identified three new benefits and one new mixed benefit/limitation under the theme “Improving care and clinical guidelines with aggregated data.” While most research focusses on the clinical, individual use of patient‐reported outcomes, we envision a more comprehensive approach that maximized the use of aggregated real‐world patient‐reported toxicity data [53]. This has the potential to enhance the “evidence ecosystem” by providing insight into daily practice and producing evidence directly from patients.

**FIGURE 2:**
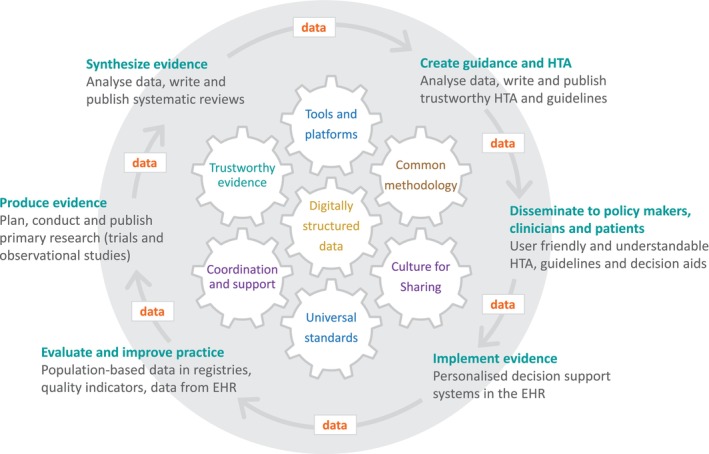
The concept of the “Digital and Trustworthy Evidence Ecosystem” visualized with the production of evidence upstream and its impact downstream (reproduced with permission [72]). HER, Electronic health records; HTA, Health technology assessment.

The evidence ecosystem functions as a feedback loop in which evidence is synthesized, guidance is created, disseminated, implemented, and evaluated—ultimately informing the production of new evidence (Figure 2) [54, 55]. This cycle can increase value and reduce waste within a true learning healthcare environment [8]. This is especially important in fields like CML care, where the focus has shifted from survival to preventing and managing chronic toxicity [56].

Although the benefits of real‐world evidence in CML care have been acknowledged [57], real‐world CML patient‐reported toxicity has not yet been systematically evaluated. Such data provide insights into treatment satisfaction and adherence [58], and the impact of toxicities on quality of life [57, 59]. Additionally, it may help address the limited stakeholder involvement and applicability of current guidelines [60]. Professionals noted that the test instrument could personalize guideline recommendations by identifying which symptoms most impact patients and by informing the development of decision aids based on this knowledge.

The fact that CML guideline developers view real‐world patient‐reported toxicity symptoms as beneficial is an important first step toward realizing their potential. However, the extent to which these benefits will be realized depends on addressing challenges such as the unstructured collection of real‐world data. Transparency will likely be key, as it enables end users to evaluate the quality and applicability of evidence resulting from real‐world data [61, 62].

To promote transparency and reproducibility, a harmonized template for evaluating real‐world evidence on treatment effects has been developed, with pilot testing underway [63]. Still, unresolved issues remain, including the frequency of data collection; integration with other databases; data audit; data ownership; electronic data capture; and the impact of the clinical pathway on data collection [64]. One potential solution is a “fit for use” assessment, which evaluates the relevance and reliability of real‐world data [62, 65]. This approach can be applied to identified knowledge gaps to help guide the collection of relevant and reliable data.

Regarding the identified novel benefit of benchmarking clinical trials and medication costs, a related concept—quality‐adjusted time without symptoms or toxicities (Q‐TWIST)—has been described in oncology [66]. However, it remains a relatively a coarse instrument, as it cannot capture real observed health‐status differences between different TKI medication regimens.

A broader application of patient‐reported toxicity data is its potential use in benchmarking medication costs, which has led to a new field of research. Intervention pharmaco‐economics uses dose‐optimization trials to reduce both toxicity and costs, a practice which has now been applied to CML care as well [67, 68, 69, 70]. Standardized real‐world patient‐reported toxicity symptom monitoring could contribute to this field by identifying interventions and/or patient groups most likely to benefit from reduced toxicity.

### 4.1 Strengths

Key strengths of this study include the application of a qualitative methodology in an under‐explored field, allowing us to capture both positive and negative perspectives with nuance. This serves as a first step toward integrating patient‐reported toxicity symptoms into guideline development. We adhered to the COREQ guidelines, conducting interviews with semi‐structured interview guides and had two researchers independently analyze the data. The analysis was based on a robust framework derived from 25 systematically sourced studies.

Another major strength is the participation of the HOVON CML‐MPN working group, which has the potential to bridge care, knowledge gaps, research, and guideline development within the evidence ecosystem. This national working group not only initiates and coordinates clinical trials but also develops guidelines, facilitating the integration of evidence into guidelines while linking knowledge gaps to new research. The Delphi procedure enabled the HOVON CML‐MPN working group to elaborate on the knowledge gaps identified during the qualitative interviews. As a result, this study translates conceptual research findings into concrete actions for CML guideline development.

### 4.2 Limitations

A limitation of our study is the underrepresentation of lower educated patients. This may have biased our results toward more active and involved patients, as those recruited through online cancer communities tend to seek more active participation compared to those recruited through population‐based methods [71]. However, several patients did indicate they would not fill out the test instrument, so we did capture the perspectives of less enthusiastic patients as well.

Additionally, three subthemes from previous studies were not identified in our research. First, the subtheme “Fuels privacy concerns” [28] may not have emerged because our study took place in a research setting with an informed consent procedure in place. Second, “Ensures holistic care” may have been less relevant in our setting, as our test instrument did not cover certain aspects of quality of life (e.g., social, spiritual factors) that were included in previous studies [17]. Third, “Inhibits interaction and rapport” may not have been identified because we did not study the test instrument in active use. Though our new limiting subtheme “Has no added value over open questions or conversation” shares some common ground, it is narrower in scope.

## 5 Conclusions

In conclusion, we identified 31 benefits and/or limitations that should be leveraged and/or addressed through targeted implementation strategies to maximize the value and uptake of systematic toxicity symptom reporting by patients. Most importantly, this study is the first to explore the views of guideline developers on real‐world toxicity symptom reporting by patients. It also identified 14 CML‐related knowledge gaps that could be addressed using such data, translating conceptual research findings into concrete actions for CML guideline development.

Further research and consensus are needed to develop coordinated methods and sustainable governance for the robust inclusion of real‐world patient‐reported toxicity data in professional guidelines. Such data will complement clinical trial findings, enhancing their applicability to real‐world practice.

## Author Contributions

Y.S., J.J.W.M.J., E.F.M.P., A.D., R.P.M.G.H., and N.M.A.B. were responsible for conception and design. Y.S. and A.C. performed interviews and wrote the manuscript. Y.S., L.V., and A.C. analyzed the data. All authors interpreted the data, gave feedback and final approval of the manuscript, had full access to all the data in the study, and had final responsibility for the decision to submit for publication.

## Conflicts of Interest

Y.S., J.J.W.M.J., E.F.M.P., A.D., R.P.M.G.H., and N.M.A.B. declare institutional research support from AbbVie, AstraZeneca, and Janssen. L.V. and A.C. have no conflicts of interest to declare. J.J.W.M.J. has received institutional research support from Novartis and BMS; honoraria to the institute from Pfizer, Novartis, and Incyte; and is the founder of Apps for Care and Science Foundation and developer of the HematologyApp. The Apps for Care and Science Foundation nonprofit organization is supported by AbbVie, AstraZeneca, Amgen, Sanofi‐Genzyme, Takeda, Jazz, Roche, Servier, BMS/Celgene, Daiichi‐Sankyo, Janssen, and Incyte. A.D. has received institutional research funding from IQVIA, speaker honoraria from Janssen and Medtronic, and is founder, shareholder, and employee of Medical Data Works B.V.

## Supporting information


Data S1.

## Data Availability

The original anonymized Dutch transcripts will be shared upon a reasonable request of researchers with a sound protocol, judged by all authors. Enquiries need to be addressed to the corresponding author. Data are available for approved analyses, immediately after publishing, for a period of 10 years. To gain access, data requestors will need to sign a data access agreement.
