# Supplementary material for: Benefits and Limitations of Real‐World Patient‐Reported Toxicity Symptom Monitoring for Guidelines and Care, as Perceived by Patients, Clinicians, and Guideline Developers
Source: Cancer Med. 2025 Apr 17;14(8):e70880. doi: 10.1002/cam4.70880 (PMC12004399; doi:10.1002/cam4.70880)
Supplement: Supplementary file 1 — Data S1. [file CAM4-14-e70880-s001.docx]

Data Supplement to:

Benefits and limitations of real-world patient-reported toxicity symptom monitoring for guidelines and care, as perceived by patients, clinicians and guideline developers

Yolba Smit, Lynn Verweij, Alexander Currie, Jeroen JWM Janssen, Eduardus FM Posthuma, André Dekker, Rosella PMG Hermens, Nicole MA Blijlevens

Contents

[COREQ checklist 2](#_Toc193189044)

[Test instrument 6](#_Toc193189045)

[Semi-structured interview guide patient focus groups or individual interviews on test instrument 6](#_Toc193189046)

[Semi-structured interview guide patient focus groups or individual interviews on graphical overview 6](#_Toc193189047)

[Semi-structured interview guide nurse practitioner focus groups or individual interviews 7](#_Toc193189048)

[Semi-structured interview guide hematologist & guideline developers focus groups or individual interviews 7](#_Toc193189049)

[Framework existing studies and this study 8](#_Toc193189050)

[Methods RAND-modified Delphi procedure 10](#_Toc193189051)

[• Questions administered in the RAND-modified Delphi procedure step (b) 10](#_Toc193189052)

[• Questions administered in the RAND-modified Delphi procedure step (c) 10](#_Toc193189053)

[• Questions administered in the RAND-modified Delphi procedure step (d) 10](#_Toc193189054)

[Themes and subthemes previously identified with illustrative quotes from this study 11](#_Toc193189055)

[References 15](#_Toc193189056)

## COREQ checklist

Table 1 COREQ (COnsolidated criteria for REporting Qualitative research) checklist^1^

| **Topic** | **Item no.** | **Guide questions/description** | **This study** |
| --- | --- | --- | --- |
| **Domain 1: Research team and reﬂexivity** | | | |
| Personal characteristics | | | |
| Interviewer/facilitator | 1 | Which author/s conducted the interview or focus group? | Yolba Smit, Alexander Currie |
| Credentials | 2 | What were the researcher’s credentials? E.g., PhD, MD | YS: MD MSc. AC: MSc |
| Occupation | 3 | What was their occupation at the time of the study? | YS: Policy advisor, PhD student. AC: Medical student |
| Gender | 4 | Was the researcher male or female? | YS: Female, AC: male |
| Experience and training | 5 | What experience or training did the researcher have? | YS: trained as a medical doctor and MSc in epidemiology. AC: trained as a medical doctor (halfway through Master phase). Both received recent training in conducting focus groups and individual interviews for qualitative research. |
| Relationship with participants | | | |
| Relationship established | 6 | Was a relationship established prior to study commencement? | YS knew two patients; one nurse-specialist and five hematologists/guideline developers form previous collaborations. One of the hematologists/guideline developer is the promotor in her PhD trajectory.AC: none. |
| Participant knowledge of the interviewer | 7 | What did the participants know about the researcher? e.g., personal goals, reasons for doing the research | All participants were made aware of the PhD position of YS and of the research questions. |
| Interviewer characteristics | 8 | What characteristics were reported about the inter viewer/facilitator? e.g., Bias, assumptions, reasons, and interests in the research topic | YS’s main interest is applied guideline development. The focus of her PhD is to evaluate how patients’ experiences can best be incorporated into the guideline development process in a systematic way. She assumes that guidelines in general can benefit to some extent from capturing patients’ experiences, such as side effects, systematically. |
| **Domain 2: Study design** | | | |
| Theoretical framework | | | |
| Methodological orientation and Theory | 9 | What methodological orientation was stated to underpin the study? e.g., grounded theory, discourse analysis, ethnography, phenomenology, content analysis | Participatory action research was the methodological orientation that underpinned this study, because it aims not only to understand reality but also improve it through change, and in doing so wants to empower the research participants^2^. |
| Participant selection | | | |
| Sampling | 10 | How were participants selected? e.g., purposive, convenience, consecutive, snowball | See Methods “Study population and recruitment”. In addition, we purposefully selected as many nurse-specialists as possible because we assumed they discuss toxicity symptoms more in depth with patients, compared to hematologists whose primary focus is disease control. When we exhausted the pool of hematology nurse-specialist, we proceeded by recruiting hematologists working in general hospitals. The number of nurse-specialists working with CML patients in the Netherlands is limited (exact figure unknown). |
| Method of approach | 11 | How were participants approached? e.g., face-to-face, telephone, mail, email | See Methods “Study population and recruitment”. In addition, We advertised for chronic phase CML patients willing to participate in either online video- or telephone-, group- or individual interviews through the website [www.cmycml.nl](http://www.cmycml.nl) and its newsletter. The call stated that we wanted to discuss the monitoring of toxicity symptoms related to TKI-treatment. We contacted all professionals face-to-face, by telephone, email, or through their professional society. |
| Sample size | 12 | How many participants were in the study? | See Table 1 and Figure 1 Flow chart of the study. |
| Non-participation | 13 | How many people refused to participate or dropped out? Reasons? | See Figure 1 Flow chart of the study. Three nurse-specialists declined participation (2 due to time pressure, one due to parental leave) and one did not respond. For the graphical overview interviews, six patients did not respond and one declined. |
| Setting | | | |
| Setting of data collection | 14 | Where was the data collected? e.g., home, clinic, workplace | For the test instrument sessions, patients were interviewed in three online focus group sessions of seven to nine participants, and three individual interviews of which one was by telephone and two online (one patient did not want to participate in a group, one patient preferred a telephone interview, and one patient for unclear reasons). For the graphical overview sessions, patients were interviewed in individual sessions (one face-to-face upon the patients request in a walk-in house near the patient’s home, and nine online video meetings). Nurse-specialists were interviewed in three individual online sessions and in two online sessions with two nurse-specialists present. Hematologists were interviewed in seven individual online sessions and two groups of two participants each. Sessions with professionals were planned as individual or group sessions according to convenience.  Participants were made aware that the test instrument was considered for routine administration within the cmyCML platform. At the start we preferred focus groups because we assumed that the interaction between participants would add valuable information. If focus groups were not practically feasible or if participants preferred individual interviews, that was possible as well. |
| Presence of non-  participants | 15 | Was anyone else present besides the participants and researchers? | A facilitator from the CMyLife team was present during all focus groups, and at two focus groups and some interviews with professionals an apprentice was present. |
| Description of sample | 16 | What are the important characteristics of the sample? e.g., demographic data, date | See Table 1 Results. |
| Data collection | | | |
| Interview guide | 17 | Were questions, prompts, guides provided by the authors? Was it pilot tested? | Semi-structured interview-guides were developed by the project group, for which the patient version was pilot tested on a patient representative. |
| Repeat interviews | 18 | Were repeat interviews carried out? If yes, how many? | No |
| Audio/visual recording | 19 | Did the research use audio or visual recording to collect the data? | See Methods “Interviews”. |
| Field notes | 20 | Were ﬁeld notes made during and/or after the interview or focus group? | Field notes were made during and/or after sessions. |
| Duration | 21 | What was the duration of the inter views or focus group? | Sessions took from 20 minutes (individual telephone interviews) to two hours (online focus groups). |
| Data saturation | 22 | Was data saturation discussed? | Saturation was defined as no new topics arising in the last two sessions, and was assessed and discussed after each session. |
| Transcripts returned | 23 | Were transcripts returned to participants for comment and/or correction? | Transcripts were not returned to participants for comments and/or corrections. The exception being that identified knowledge gaps entered the RAND-modified Delphi procedure, in which eight out of ten experts had been interviewed themselves. |
| **Domain 3: analysis and ﬁndings** | | | |
| Data analysis | | | |
| Number of data coders | 24 | How many data coders coded the data? | Two. |
| Description of the coding tree | 25 | Did authors provide a description of the coding tree? | Table 2 Results section. |
| Derivation of themes | 26 | Were themes identiﬁed in advance or derived from the data? | See Methods “Data analysis”. |
| Software | 27 | What software, if applicable, was used to manage the data? | See Methods “Data analysis”. |
| Participant checking | 28 | Did participants provide feedback on the ﬁndings? | No, except for feedback on the knowledge gaps identified, through the RAND-modified Delphi procedure. |
| Reporting | | | |
| Quotations presented | 29 | Were participant quotations presented to illustrate the themes/ﬁndings? Was each quotation identiﬁed? e.g., participant number | Table 2 in Results and Table 2 in Supplement. |
| Data and ﬁndings consistent | 30 | Was there consistency between the data presented and the ﬁndings? | Results section. |
| Clarity of major themes | 31 | Were major themes clearly presented in the ﬁndings? | Table 2, Results section. |
| Clarity of minor themes | 32 | Is there a description of diverse cases or discussion of minor themes? | Results and Discussion sections. |

## Test instrument

We constructed a 48-item instrument out of all symptom items taken from the EORTC QLQ-CML-24 plus all CML symptoms from the EORTC Symptom Set^3-5^. During development and validation phases (not reported on here) the instrument grew to 61 items, the EORTC IL196.

Table 2 The 61 items included in the test instrument (EORTC IL196) at the end of the study

| Skin problems | Feeling weak | Unintentional gas/flatulence |
| --- | --- | --- |
| Skin colour change | Lacking energy | Pain |
| Itchy skin | Feeling drowsy | Pain interference |
| Skin rash | Trouble sleeping | Pain in chest |
| Dry/flaking/cracked skin | Headaches | Muscle cramps |
| Sore/painful skin | Dizziness | Aches/pains muscles/joints |
| Easy bruising | Difficulty remembering | Muscle weakness |
| Hair loss | Difficulty concentrating | Swelling body parts |
| Eye problems | Trouble thinking clear | Tingling/numbness hands/feet |
| Watery eyes | Feeling depressed | Shortness of breath |
| Burning eyes | Feeling irritable | Coughing problems |
| Discomfort eyes bright light | Feeling tense | Fevers/chills |
| Blurred vision | Worrying | Excessive sweating |
| Swelling face/eyes | Frequent urination | Problems tolerating heat/cold |
| Hearing problems | Bloated feeling abdomen | Hot flushes |
| Dry mouth | Acid indigestion/heartburn | Sore/enlarged nipples/breasts |
| Pain/soreness mouth | Feeling nauseated | Less interest sex |
| Different taste food/drink | Vomiting | Less sexual enjoyment |
| Lack appetite | Diarrhoea | Difficulty getting/maintaining erection |
| Tiredness | Feeling constipated |  |
| Needing to rest | Abdominal pains/cramps |  |

## Semi-structured interview guide patient focus groups or individual interviews on test instrument

- Prompts:
  - Can you elaborate?
  - Can you give me an example?
  - How do you feel about [example given by other patients]?
- What do you want to know about your side effects?
- Would you use this questionnaire to keep score of your side effects? Why?
- What insights would the answers to the questionnaire give you, or to what insights might repeated answers lead?
- To which outcomes or actions would these insights lead?
- Would you like to compare your answers to your own previous answers, or to other CML patients, or otherwise?

## Semi-structured interview guide patient focus groups or individual interviews on graphical overview

- What is your opinion on the graphical overview?
- What were your first thoughts?
- What do you think of the design?
  - In general, legend, graphs, icons
  - What can be improved and how?
  - What could be left out?
  - What could be added?
- The symptoms that you personally scored ‘not at all’ have been left out. What is your opinion on that?
- Some professionals stated they would like patients to indicate which symptoms they want to discuss during consultation. What do you think?
- How does the graphical overview influence your wellbeing?
  - Experiencing symptoms?
  - Change in anxiety, stress?
  - Anything else?
- Would you like to discuss your overview with others? If yes, with whom and why? If not, why not?

## Semi-structured interview guide nurse practitioner focus groups or individual interviews

- Prompts:
  - Can you elaborate?
  - Can you give me an example?
  - How do you feel about [example given in another workshop]?
- The answers to the questions in the questionnaire might be useful during consultations. What is your opinion on that?
- What would you do with the information from the questionnaire?
- Would you discuss the information during consultation? Why yes/no?
- Would you also like the information from the questionnaire if patients switched TKI, or changed TKI dosage, or stopped their TKI?
- How can we reach patients with low health literacy when we want to use this questionnaire?

## Semi-structured interview guide hematologist & guideline developers focus groups or individual interviews

In addition to above, questions relevant to the CML guideline were:

- As a guideline developer/hematologist, what is your opinion on the collection of symptoms of side effects of CML patients?
- Will the information benefit the guideline? Why yes/no?
- Which evidence gaps might this information help solve?
- Are there advantages/disadvantages to using this instrument, when developing the guideline?
- Are there requirements for the collection of symptom data for guideline development?
- Would information on the symptoms of patients that stopped their TKI be useful for the guideline?

## Framework existing studies and this study

Table 3 Framework of previous studies and this study, with new (sub)themes and their assessment in italics

| **Previous studies^6-30^** | | **This study** |
| --- | --- | --- |
| Active patient involvement and partnership | | Idem |
| - Enables greater awareness and reflection (Benefit) | | - Idem |
| - Encourages patient involvement (Benefit) | | - Idem |
| - Facilitates goal setting and shared decision making (Benefit) | | - Idem |
| - Influences honesty (Mixed) | | - Idem |
| - Permits discussion of sensitive topics (Benefit) | | - Idem |
|  | | - Enables *self-prevention* and self-care (Benefit) ^£^ |
| - Leads to worse symptom experience (Limitation) | | - Idem |
|  | Fulfils desire to help others (Benefit) | - Idem |
|  | Fuels privacy concerns (Limitation) | - Not identified - *Objectifies subjective experience (Benefit)* |
| Focus of consultation | | Idem |
| - Helpful as a screening tool (Benefit) | | - Idem |
| - Prioritizes patients’ needs (Benefit) | | - Idem |
| - Provides one piece of the picture (Limitation) | | - Idem |
| - Structures consultations and improves efficiency (Benefit) | | - Idem |
| - Shifts away from the main medical problem (Limitation) ^$^ | | - *Suits paramedical consultation (Mixed) ^$^* |
| - Raises unrealistic expectations for care (Limitation) ^$^ | | - Merged with ‘Suits paramedical consultation’ |
|  | | - Provides redundant information^§^ |
|  | | - Provides reassurance that clinicians care^¥^ |
| Quality of care | | Quality of individual patient care |
| - Assists diagnosis and enables tailored (self-)care (Benefit) ^£^ | | - Moved to ‘Active patient involvement and partnership’. Care part covered by ‘Helps determine and monitor side effects’ and ‘Prompts appropriate, standardized action and acceptance’ |
| - Ensures holistic care (Benefit) | | - Not identified |
| - Can inaccurately estimate the problem (Limitation) | | - *…* /not specific enough to be clinically meaningful ^#^ |
| - Prompts appropriate, standardized action (Benefit) | | - … *and acceptance* |
| - Is an opportunity for the education of patients and professionals (Benefit) | | - Assists in learning (Benefit) |
|  | | - Helps determine *and monitor* side effects of treatment * |
|  | | - *Does not establish a causal link (Limitation)* |
| Standardized monitoring of patient outcomes over time | | Moved to ‘Quality of individual patient care’ |
| - Helps determine effectiveness and side-effects of treatment (Benefit) | | - Merged, moved to ‘Quality of individual patient care’ * |
| - Useful for monitoring changes and tracking progress (Benefit) | | - Merged, moved to ‘Quality of individual patient care’ * |
| Patient-clinician relationship | | Moved or not identified |
| - Provides reassurance that clinicians care (Benefit) | | - Moved to ‘Focus of consultation’ ^¥^ |
| - Inhibits interaction and rapport (Limitation) | | - Not identified |
| Lack of valuable information | | Merged/moved to other themes |
| - PRO data is not specific enough to be clinically meaningful (Limitation) | | - Merged with ‘Can inaccurately estimate the problem’ ^#^ |
| - Provides redundant information (Limitation) | | - Moved to ‘Focus of consultation’^§^ |
| Suitability for all patients | | Suitability and acceptability |
| - Suitability for all patients (Limitation) | | *…, clinicians and workflows* |
| - Confronts too much with disease (Limitation) | | - Confronts too much with disease (Limitation) |
|  | | - *Usefulness depends on (change in) symptom severity (Mixed)* |
|  | | - *Has no added value over open questions or conversation (Limitation)* |
| Improving care with aggregated data | | Improving care and clinical guidelines with aggregated data |
| - Data interpretation is challenging (Limitation) | | - *Real-world data has pros and cons (Mixed)* |
| - Benchmarks hospital performance and clinical trials which will eventually lead to lower costs (Benefit) | | - Benchmarks clinical trials and medication costs (Benefit) |
| - Puts individual experience in context (Benefit) | | - Puts individual experiences in context (Benefit) |
|  | | - *Systematically includes patients’ experiences in guidelines (Benefit)* - *Personalizes guideline advise (Benefit)* |
|  | | - *Fills in knowledge gaps (Benefit)* |

Main themes and subthemes were taken from Campbell et al.^6^ and completed with (sub)themes from other relevant studies. ‘Idem’ indicates that we used the exact same phrasing for the (sub)theme as previous studies did. Special characters (^£^, ^$^, *, ^#^, ^§^, ^¥^) indicate merged with, or moved to other (sub)themes

## Methods RAND-modified Delphi procedure

We used a RAND-modified Delphi method to reach consensus on, and prioritize knowledge gaps on TKI toxicity, that may be addressed (in part) by aggregated data from the test instrument^31^. In step (1) all knowledge gaps mentioned during the interviews were identified. For step (2) all panel members received a questionnaire by email, asking after completeness of, and agreement with, the formulated knowledge gaps (see the questionnaires below). All panel members had the opportunity to add new knowledge gaps and to formulate feedback. Answers were analysed by adding all new knowledge gaps and by reformulating knowledge gaps, based on the feedback received. For step (3) the set was sent to panel members for final appraisal, including the feedback received and how feedback was processed. In step (4) panel members were asked to prioritize knowledge gaps in the final set, in order of relevance for the Dutch CML guideline.

## Questions administered in the RAND-modified Delphi procedure step (b)

1. Which knowledge gaps are you missing in this list of nine knowledge gaps, that you consider relevant for the CML guideline and that could be (partly) answered with patient-reported TKI-related toxicity?
2. Which of the nine knowledge gap(s) can be better formulated, and in what way?
3. Do you have general feedback regarding these knowledge gaps?

## Questions administered in the RAND-modified Delphi procedure step (c)

1. Is the feedback properly processed?
2. Do you have any feedback on the knowledge gaps as they are currently formulated?

## Questions administered in the RAND-modified Delphi procedure step (d)

Please indicate below what priority you think each relevant knowledge gap has

- Enter a number between 1 (highest priority) and 14 (lowest priority) behind each knowledge gap
- Use each number from 1 to 14 only once

## Themes and subthemes previously identified with illustrative quotes from this study

Table 4 Themes and subthemes previously identified*, with an assessment of benefit or limitation, with illustrative quotes from this study

| **Themes and subthemes** | **Benefit or limitation** | **Illustrative quotes** |
| --- | --- | --- |
| Active patient involvement and partnership | | |
| Enables greater awareness and reflection | Benefit | So, seeing a list like this helps to be able to identify what symptoms are, or could indeed possibly be, CML related, before I sweep them under the carpet or put them in a drawer and don’t want to know anything more about them (P)  Maybe there are things that don’t, yes, that don’t come to my mind or that I don’t think about, that I might have to tick (P)  Yes, that’s possible, look, I, I keep track of my own side effects and then I try to relate those to things I have done or that I do (P)  So, I have tried for myself to uhm get in a sort of biological rhythm in taking the medication […] to see what fits me best. And that’s why I’ve kept notes of all my side effects, to come to my ideal situation (P) |
| Encourages patient involvement | Benefit | I think it is also a good summary for them [patients] to prepare, for the outpatient clinic, or for when they speak to us [nurse-specialists] or a specialist (NS)  I can also imagine that it is nice for patients, so, okay, the scores are high so I will talk about that in the doctor’s office (H/GD)  Well, I do think that if, for example, some items keep popping up that really trouble you, then you can bring it to your hematologist and say: “Well, listen, this or that bothers me for such a long time, can we do anything about it?” (P) |
| Facilitates goal setting and shared decision making | Benefit | Well look, the challenge is more: the extent to which the information provided by the patients PROs can influence decision-making in the doctor's office. A good response [to treatment] with as few side effects as possible, that is the trick. And the normal guidelines are focused on the best possible response. And side effects are not discounted there. While you can also opt for a slightly less optimal response, but with less misery and a better quality of life (H/GD) |
| Influences honesty | Limitation | If you have a patient who says yes to everything, then I don't do anything with it. […] You can never have everything. […] you have patients who supposedly suffer from everything. And you must get that from a conversation and not from a questionnaire. There are people who […] do not dare to say that it is going well, because otherwise they don't feel heard. […] I always find that the complicated part of these kinds of questionnaires. That you have no subjective feeling about whether this is realistic (H/GD)  I used to really work a lot [with questionnaires] and then I would ask my open question and something different always emerged. […] So that I thought: “oh yes but why do you still fill this in if you give me completely different answers?” So, I would keep asking my open question. […] So, for me open questions give me way more than that list for those people, but I don't know why that is (NS) |
| Permits discussion of sensitive topics | Benefit | But, sometimes uhm, I think gosh, I must defend myself by saying, uhm, guys, the treatment is there and working great, but uhm, can we talk side effects because uhm they do influence quality of live negatively to a great extent. And uhm, I often feel guilty talking about this with people, or I think oh, I must defend myself (P)  What I really do miss, particularly about symptoms they [patients] want to discuss, is sexual dysfunction. [..] While that is a really complex topic for a lot of people to discuss (NS) |
| Leads to worse symptom experience | Limitation | I actually started feeling everything (NS)  But they [the questions] are negatively formulated […] they are not positively formulated in the context of positive health (H/GD) |
| Fulfils desire to help others | Benefit | I wouldn’t fill it in for me, but for others. […] to, to, just like this, help them to gain insight […] I would like to help, uhm, others (P) |
| Focus of consultation |  |  |
| Helpful as a screening tool | Benefit | Look, I see this as a screening instrument, right? […] So, you check, are there things that have not been reported spontaneously that are relevant, and uhm, if a flag is raised, so, somebody experiences a symptom than you go into that, checking if there is a relationship at all and continue questioning the impact (H/GD)  The medical doctor must see it when it isn’t stated that action is still needed […] so I think more like prior notice (H/GD) |
| Prioritizes patients' needs | Benefit | Well, I think it is especially useful at the time when you, uhm discuss with your treating physician, that they get a better insight in whether you are doing fine in the long term (P)  For the physician, the main issue is whether the disease is under control, uhm, and also a little bit about quality of life, but no, not a lot, because that is not core business, so, at that moment I think (P)  If a doctor doesn’t want to hear about it, that whining, that the patient can say: “yes, but look, it’s very important to me because this is a complaint that comes back every time” (H/GD)  So, the patient [gets] the possibility to discuss it [their symptoms] (H/GD) |
| Provides reassurance that clinicians care | Benefit | It is certainly a starting point to talk about with a healthcare provider. If I tick a lot, the [specialist] might say: okay, it's worse than I thought. And you also come to talk about quality of life. So, you can discuss: okay, are there things we can do? […] So, you can address that better that way. Then she [nurse-specialist] also has a little insight into how I experience it (P) |
| Provides one piece of the picture | Limitation | But that's the problem with questionnaires, you don't ask everything, and you might miss things (H/GD) |
| Structures consultations and improves efficiency | Benefit | Well, I think it is especially useful when you talk to your treating physician, that he gains a bit more insight in whether you are doing well in the long term. It’s often only 10 minutes you get with him […] before you know it, you’re outside again (P)  And in our hospital, I see that in the anaesthesia department, they do that very well, they send all questionnaires to patients preoperatively and that works great. That improves the consultation throughput, right? So, in the end, uhm, it provides a benefit for health care (NS)  And […] shorten contact time. Or even, in my ideal situation, skip it, you know. What I want is on demand consultations. So, if you fill out the [test] instrument, and I see it and its’s stable, and you have no further questions, then we can skip it the next three to four months. Also, just because, as you [other participant] just said, not focus on the disease too much (NS) |
| Provides redundant information | Limitation | As a researcher I want to know as much as possible, as a medical doctor I do not want to know everything. Because I can’t do anything about it, I can’t … then I think, well, all those symptoms … (H/GD)  It's a bit more my personal feeling. Like: “gee, what are we going to do with this data? What does it lead to? Does it really have added value?.” On the other hand, if you have a decent conversation with a patient, a lot of things are already discussed (H/GD)  But I don't really need to know everything that bothers patients just a little bit. […] and this is not such a politically correct comment, but I have fifteen minutes for them, and I see them 3 times a year or so, and I want to know the highlights […] then I really just want to know the highlights and know what really bothers them (H/GD)  Well look, […] I usually do outpatients, patients who are involved in all kinds of things and have to deal with all kinds of things, and this provides so much information that I think: well, I ask 6 questions: are you tired, do you have muscle problems, do you have problems with your skin, do you have problems with your eyes, how are things going in your private life and are there any changes, can I help you with anything? ? Then I also get the information I need. Then I think this is just too much stimulus, too much information (H/GD) |
| Quality of *individual* *patient* care | | |
| Assists in learning | Benefit | Yes, then I’m looking for how it is for others. I try to learn from that, I find it interesting […] let’s say 100 people keep track of all [their] side effects, then a certain profile emerges, well, I find that interesting. Because that can help me, but maybe others too (P)  Indeed, to start recognizing patterns [P]  What I would find nice myself is if there are people who, when you get the data, yes, I always have such and such a side effect, but this works great. And that I didn't know that yet [H/GD] |
| Can inaccurately estimate the problem/not specific enough to be clinically meaningful | Limitation | Because were one person fills out ‘a little’, the other person states ‘quite a bit’, which maybe has more to do with personality than with the severity of the itch (NS)  So, because, if I’m a squeaker, I mind everything and if I’m a tough boy, I don’t mind at all (H/GD)  Are you short of breath? […] “Well, yesterday it was muggy. […] So, if the humidity is over 80%, I feel a tightness in my chest, but I am not short of breath” (H/GD)  Al lot of patients indicate they are tired all the time, but the persons I see have aged 10 years in the meantime, do you understand? So, uhm, age moves up. So, age is crucial and the interpretation of symptoms […] [is] different for women than for men (H/GD)  Do you ever feel short of breath? Yeah well, as I walked up the stairs this morning, I thought, oh god, I have to work again, oh, I'm tired. Do you understand? (H/GD) |
| Suitability and acceptability |  |  |
| Confronts too much with disease | Limitation | And not that it doesn’t matter, or that I can’t think about it [i.e., symptoms of toxicity], but yes, I prefer not to think about it (P)  That they [patients] say: “yes, I kind of stopped because I was confronted too much with my symptoms. I started to feel everything” So that’s the downside. So that is also the freedom that patients should have, of whether or not to use [the questionnaire] (H/GD)  The complicated thing is that you start thinking about everything. That's my experience too. When a doctor asks me something, I think, 'Oh, do I have it or don't I?' And then I say no, for example, but then I think, 'But do I really never have that?' While the question would never have occurred to me if it hadn't been an issue. […] burdened with questions about what is possible. That's like reading the package leaflet, you know? Then you are waiting to get all those things, while that may not apply to you at all (P) |
| Improving care and clinical guidelines with aggregated data | | |
| Benchmarks clinical trials and medication costs | Benefit | You know that perspective can be brought forward by saying, yes, but in practice you see that this is the trouble, you know, the burden which this medication causes (H/GD)  And especially because this is a disease that [people] carry with them for the rest of their lives and need to take medication for the rest of their lives, so are condemned the rest of their lives, I think, we shouldn’t just look at maximum disease response, so normal survival […] so that perspective needs to be taken into account by saying, yes, well, this is the burden in real-world from this medication. And that, I think, needs to be assessed scientifically, how bad that burden is, and then relate that to uhm, uhm, response, and then weigh (H/GD)  […] has a relationship with financial toxicity, in that uhm, we pay too much for the medication […] because something is promised with a certain response […] but that ignores toxicity and impact on [quality] of life, and that is not discounted in the price we must pay (H/GD) |
| Puts individual experience in context | Benefit | In the discussion with the patient, that's when the benchmark comes into play [H/GD]  Do you know what they have for CLL [chronic lymphoid leukemia]? Uhm uh, they have bars for, well, your own complaints, and uh there is a bar next to it, and that is your peer group, namely people with CLL, and there is a bar next to that for the, well, normal population. That is also very reassuring […] because then you have a lot of people who say yes: I am tired, but it's actually not too bad for me because those other people suffer much more [H/GD] |

* Main themes and subthemes were taken from Campbell et al.^6^ and completed with (sub)themes from other relevant studies^7-30^. Identifiers in brackets after quotes: H/GD: hematologist/guideline developer; NS: nurse-specialist; P: patient

## References

1 Tong A, Sainsbury P, Craig J. Consolidated criteria for reporting qualitative research (COREQ): a 32-item checklist for interviews and focus groups. Int J Qual Health Care 2007; 19 (6): 349-357.

2 Baum F, MacDougall C, Smith D. Participatory action research. J Epidemiol Community Health 2006; 60 (10): 854-857.

3 Efficace F, Baccarani M, Breccia M et al. International development of an EORTC questionnaire for assessing health-related quality of life in chronic myeloid leukemia patients: the EORTC QLQ-CML24. Qual Life Res 2014; 23 (3): 825-836.

4 Efficace F, Iurlo A, Patriarca A et al. Validation and reference values of the EORTC QLQ-CML24 questionnaire to assess health-related quality of life in patients with chronic myeloid leukemia. Leuk Lymphoma 2021; 62 (3): 669-678.

5 Sodergren SC, Wheelwright SJ, Fitzsimmons D et al. Developing Symptom Lists for People with Cancer Treated with Targeted Therapies. Targeted Oncology 2021; 16 (1): 95-107.

6 Campbell R, Ju A, King MT, Rutherford C. Perceived benefits and limitations of using patient-reported outcome measures in clinical practice with individual patients: a systematic review of qualitative studies. Qual Life Res 2021.

7 Boyce MB, Browne JP, Greenhalgh J. The experiences of professionals with using information from patient-reported outcome measures to improve the quality of healthcare: a systematic review of qualitative research. BMJ Qual Saf 2014; 23 (6): 508-518.

8 Carfora L, Foley CM, Hagi-Diakou P et al. Patients' experiences and perspectives of patient-reported outcome measures in clinical care: A systematic review and qualitative meta-synthesis. PLoS One 2022; 17 (4): e0267030.

9 Glenwright BG, Simmich J, Cottrell M et al. Facilitators and barriers to implementing electronic patient-reported outcome and experience measures in a health care setting: a systematic review. J Patient Rep Outcomes 2023; 7 (1): 13.

10 Greenhalgh J, Dalkin S, Gooding K et al. Functionality and feedback: a realist synthesis of the collation, interpretation and utilisation of patient-reported outcome measures data to improve patient care. Southampton (UK)2017.

11 Nic Giolla Easpaig B, Tran Y, Bierbaum M et al. What are the attitudes of health professionals regarding patient reported outcome measures (PROMs) in oncology practice? A mixed-method synthesis of the qualitative evidence. BMC Health Serv Res 2020; 20 (1): 102.

12 Bennink C, de Mul M, van der Klift M et al. Improving Outcome-Driven Care in Multiple Myeloma Using Patient-Reported Outcomes: A Qualitative Evaluation Study. Patient 2023; 16 (3): 255-264.

13 Lai-Kwon J, Rutherford C, Jefford M et al. Using Implementation Science Frameworks to Guide the Use of Electronic Patient-Reported Outcome Symptom Monitoring in Routine Cancer Care. JCO Oncol Pract 2024.

14 Lombi L, Alfieri S, Brunelli C. 'Why should I fill out this questionnaire?' A qualitative study of cancer patients' perspectives on the integration of e-PROMs in routine clinical care. Eur J Oncol Nurs 2023; 63: 102283.

15 Sandhu S, King Z, Wong M et al. Implementation of Electronic Patient-Reported Outcomes in Routine Cancer Care at an Academic Center: Identifying Opportunities and Challenges. JCO Oncol Pract 2020; 16 (11): e1255-e1263.

16 Tolstrup LK, Pappot H, Bastholt L et al. Patient-Reported Outcomes During Immunotherapy for Metastatic Melanoma: Mixed Methods Study of Patients' and Clinicians' Experiences. J Med Internet Res 2020; 22 (4): e14896.

17 Tran C, Dicker A, Leiby B et al. Utilizing Digital Health to Collect Electronic Patient-Reported Outcomes in Prostate Cancer: Single-Arm Pilot Trial. J Med Internet Res 2020; 22 (3): e12689.

18 Smith TG, Beckwitt AE, van de Poll-Franse LV et al. Oncology team perspectives on distress screening: a multisite study of a well-established use of patient-reported outcomes for clinical assessment. Support Care Cancer 2022; 30 (2): 1261-1271.

19 Brooks JV, Poague C, Formagini T et al. The Role of a Symptom Assessment Tool in Shaping Patient-Physician Communication in Palliative Care. J Pain Symptom Manage 2020; 59 (1): 30-38.

20 Garcia Farina E, Rowell J, Revette A et al. Barriers to Electronic Patient-Reported Outcome Measurement Among Patients with Cancer and Limited English Proficiency. JAMA Netw Open 2022; 5 (7): e2223898.

21 Govindaraj R, Agar M, Currow D, Luckett T. Assessing Patient-Reported Outcomes in Routine Cancer Clinical Care Using Electronic Administration and Telehealth Technologies: Realist Synthesis of Potential Mechanisms for Improving Health Outcomes. J Med Internet Res 2023; 25: e48483.

22 Kennedy F, Shearsmith L, Holmes M et al. 'We do need to keep some human touch'-Patient and clinician experiences of ovarian cancer follow-up and the potential for an electronic patient-reported outcome pathway: A qualitative interview study. Eur J Cancer Care (Engl) 2022; 31 (2): e13557.

23 Lapen K, Sabol C, Tin AL et al. Development and Pilot Implementation of a Remote Monitoring System for Acute Toxicity Using Electronic Patient-Reported Outcomes for Patients Undergoing Radiation Therapy for Breast Cancer. Int J Radiat Oncol Biol Phys 2021; 111 (4): 979-991.

24 McCready TM, Stabile C, Vickers A et al. A Remote Symptom Monitoring Tool As Part of Ambulatory Cancer Surgery Recovery: A Qualitative Analysis of Patient Experience. JCO Oncol Pract 2023; 19 (8): 595-601.

25 Pompili C, Boele F, Absolom K et al. Patients' views of routine quality of life assessment following a diagnosis of early-stage non-small cell lung cancer. Interact Cardiovasc Thorac Surg 2020; 31 (3): 324-330.

26 Samuel CA, Smith AB, Elkins W et al. Racial differences in user experiences and perceived value of electronic symptom monitoring in a cohort of black and white bladder and prostate cancer patients. Qual Life Res 2021; 30 (11): 3213-3227.

27 Shunmugasundaram C, Sundaresan P, White K et al. Development and implementation barriers of a new patient-reported measure: The Radiation therapy-related Inconvenience Questionnaire (RIQ). J Med Imaging Radiat Oncol 2023; 67 (7): 777-788.

28 Skåre TS, Midtbust MH, Lund J et al. Barriers and Facilitators When Implementing Electronic Patient-Reported Outcome Measures at a Municipal Cancer Care Unit: A Qualitative Study. Cancer Nurs 2023; 46 (4): E268-e275.

29 Snyder C, Hannum SM, White S et al. A PRO-cision medicine intervention to personalize cancer care using patient-reported outcomes: intervention development and feasibility-testing. Qual Life Res 2022; 31 (8): 2341-2355.

30 Sung JH, Brown MC, Perez-Cosio A et al. Acceptability and accuracy of patient-reported outcome measures (PROMs) for surveillance of breathlessness in routine lung cancer care: A mixed-method study. Lung Cancer 2020; 147: 1-11.

31 Dalkey N, Helmer O. An experimental application of the delphi method to the use of experts. Management Science 1963; 9 (3): 458-467.
